# High triglyceride–glucose index and changes in triglyceride–glucose index are associated with an increased risk of hypogonadism in middle-aged Taiwanese men: a retrospective longitudinal study

**DOI:** 10.3389/fendo.2026.1793890

**Published:** 2026-05-14

**Authors:** Chun-Cheng Liao, Chia-Lien Hung, Jane-Dar Lee, Yu-Juei Hsu, Chih-Yuan Lin, Yu-Lueng Shih, Shih-Hung Tsai

**Affiliations:** 1 Department of Family Medicine, Taichung Armed Forces General Hospital, Taichung, Taiwan; 2 Department of Medical Education and Research, Taichung Armed Forces General Hospital, Taichung, Taiwan; 3 School of Medicine, National Defense Medical University, Taipei, Taiwan; 4 Graduate Institute of Public Health, College of Public Health, National Defense Medical University, Taipei, Taiwan; 5 Division of Urology, Department of Surgery, Taichung Armed Forces General Hospital, Taichung, Taiwan; 6 Division of Nephrology, Department of Internal Medicine, Tri-Service General Hospital, National Defense Medical University, Taipei, Taiwan; 7 Tri-Service General Hospital Songshan Branch, Taipei, Taiwan; 8 Division of Cardiovascular Surgery, Department of Surgery, Tri-Service General Hospital, National Defense Medical University, Taipei, Taiwan; 9 Taichung Armed Forces General Hospital, Taichung, Taiwan; 10 Division of Gastroenterology, Department of Internal Medicine, Tri-Service General Hospital, National Defense Medical University, Taipei, Taiwan; 11 Department of Emergency Medicine, Tri-Service General Hospital, National Defense Medical University, Taipei, Taiwan; 12 Department of Physiology and Biophysics, Graduate Institute of Physiology, National Defense Medical University, Taipei, Taiwan

**Keywords:** free testosterone, hypogonadism, middle age Taiwanese men, total testosterone, triglyceride–glucose index

## Abstract

**Purpose:**

The triglyceride–glucose (TyG) index is a well-established marker of metabolic and cardiovascular risk. Although prior research has identified inverse associations between the TyG index and androgen-related health—including testosterone levels and erectile dysfunction—there is limited data on how longitudinal changes in this index correlate with hormonal shifts. Therefore, this study investigated the association between dynamic changes in the TyG index and total/free testosterone levels within a retrospective Taiwanese cohort to evaluate its utility in identifying men at risk for androgen deficiency.

**Materials and methods:**

Data were obtained from a longitudinal MJ Health Screening Center database in Taiwan (2011–2022), comprising repeated health examination records of 4,269 men aged 45–65 years. Collected variables included demographic characteristics, triglyceride, fasting sugar, TyG index, and total testosterone. To account for within-subject correlations in the repeated measurements, generalized estimating equations (GEEs) were employed using multivariate models to analyze the associations between TG/FG phenotypes, TyG index, and hypogonadism.

**Results:**

Among 4,269 men aged 45–65 years (7,268 records), 11.2% had hypogonadism. After adjustment for measurement time, age, body mass index, waist circumference, blood pressure, and high-density lipoprotein, the TyG index was significantly associated with increased hypogonadism risk (odds ratio [OR] = 1.90; 95% confidence interval [CI], 1.58–2.28; p < 0.001) and inversely with free testosterone (β = −0.294, p = 0.005). Compared with the normal fasting glucose and normal triglycerides (NFGNT) group, the Hypertriglyceridemic fasting glycemia (HTFG) group exhibited the highest risk (OR = 2.55) and the greatest reduction in free testosterone (β = −0.530), followed by the high triglyceride (HTG) group (OR = 2.16; β = −0.465) and the elevated fasting glucose (EFG) group (OR = 1.61; β = −0.254). In men with baseline testosterone ≥300 ng/dL, hypogonadism risk increased up to 2.38-fold when the TyG index increased by ≥0.5.”

**Conclusions:**

In conclusion, the TyG index serves as a valuable tool for identifying men at risk of androgen deficiency. Longitudinal increases in this index are independently associated with an elevated risk of hypogonadism and declining free testosterone levels, with the high triglyceride with high fasting glucose (HTFG) group exhibiting the most pronounced risk.

## 1 Introduction

Insulin resistance (IR) is a fundamental mechanism underlying type 2 diabetes mellitus (T2DM), obesity, dyslipidemia, and cardiovascular disease (CVD). Recent systematic reviews have indicated that IR is a burgeoning global health concern, with a global prevalence of 26.53% and estimates ranging between 26% and 30% across different populations and geographical regions (1). Chronic IR induces hyperinsulinemia, lipid accumulation, and systemic inflammation, promoting the progression of atherosclerosis and metabolic syndrome (2–4). The triglyceride–glucose (TyG) index, calculated from fasting TG and glucose levels, has been validated as a simple and reliable surrogate marker of IR, comparable with the homeostatic model assessment (HOMA-IR) and the euglycemic–hyperinsulinemic clamp (5, 6). Elevated TyG index values and related indices (e.g., TyG–body mass index [BMI] and TyG–waist circumference [WC]) are strongly associated with metabolic syndrome, incident diabetes, cardiovascular events, and increased cardiovascular–kidney–metabolic risk (7–10). Given its accessibility and stability, the TyG index is considered a useful marker of long-term metabolic burden and cardiometabolic risk.

Male hypogonadism, characterized by reduced testosterone secretion, is increasingly recognized as part of the spectrum of metabolic disorders and is closely associated with IR, visceral adiposity, and cardiovascular risk (11–14). Epidemiological research, such as the HIM study, has reported that the prevalence of hypogonadism (defined as TT < 300 ng/dL) among men aged 45 years or older is as high as 38.7% (15). Testosterone promotes lean mass and insulin sensitivity; thus, its deficiency contributes to visceral fat accumulation, inflammation, and impaired glucose metabolism, perpetuating the cycle between metabolic dysfunction and Hypogonadism (16).

Recent evidence suggests that visceral fat plays a crucial role in mediating the relationship between low testosterone and metabolic risk (17). Several studies have also reported that visceral obesity is associated with low testosterone levels, male infertility, and erectile dysfunction (17, 18). Testosterone deficiency can increase lipoprotein lipase activity and facilitate fatty acid uptake by adipocytes, promoting visceral fat accumulation and sustaining metabolic syndrome features such as hypertriglyceridemia and IR (16). Additionally, higher TyG index values, indicative of IR, are associated with lower testosterone levels and a higher prevalence of hypogonadism in men with T2DM, with some evidence suggesting that TyG may strongly correlate with hypogonadism than HOMA-IR (19, 20).

However, most available studies are cross-sectional; therefore, it remains unclear whether longitudinal changes in the TyG index predict the development of hypogonadism. To address this knowledge gap, this study aimed to determine whether longitudinal change in TyG index is associated with the risk of incident hypogonadism among initially eugonadal men. Clarifying this association may help establish the TyG index as a practical biomarker linking IR, visceral adiposity, and hypogonadism. Given the unique metabolic characteristics of East Asian populations, including a higher propensity for insulin resistance at relatively lower body mass index levels, population-specific investigations are warranted. In addition, the MJ Health database provides longitudinal data with repeated measurements, enabling a more robust evaluation of temporal associations compared with cross-sectional datasets.

## 2 Materials and methods

### 2.1 Study design, area, and population

This retrospective longitudinal study was conducted using repeated health examination data from the MJ Health Screening Center database (2011–2022). The center is a premier private health screening institution in Taiwan, providing comprehensive, self-paid health examinations across major metropolitan regions with costs ranging from USD $200 to $730. While the participant demographic is generally representative of the Taiwanese population, it may reflect a cohort with higher-than-average health awareness.

For this analysis, a total of 4,269 male participants were initially identified, regardless of baseline medical conditions. A consecutive sampling approach was used, including all eligible male participants from the MJ Health Screening Center database during the study period. Based on a *post hoc* power analysis for GEE models, this sample size yields a statistical power > 0.99 for detecting small effect sizes (f^2^ = 0.02) at an alpha level of 0.05, ensuring robustness of multivariate analysis and minimizing the risk of Type II errors. To ensure data integrity, we excluded individuals with missing values for key variables, specifically total testosterone (TT) and free testosterone (FT). Additionally, the study was restricted to middle-aged and older men, excluding those aged outside the range of 45 to 65 years.

### 2.2 Data collection and laboratory procedure

For this study, retrospective data were obtained and extracted from the MJ Health Research Foundation database using a standardized data extraction protocol and a structured electronic request form to ensure data integrity and consistency. The extracted dataset included comprehensive health information, such as demographic characteristics, anthropometric assessments (BMI and waist circumference), and biochemical profiles (including fasting glucose, lipid levels, and sex hormones). These procedures were conducted under the governance of the MJ Health Research Foundation to maintain de-identification and participant privacy. All participants underwent standardized physical examinations and anthropometric assessments. Body mass index (BMI) was calculated as weight in kilograms divided by the square of height in meters (kg/m^2^). Waist circumference (WC) was measured at the midpoint between the iliac crest and the lower rib, with participants standing and fully exhaling. To ensure systematic laboratory accuracy, blood samples were obtained after an overnight fast. At the MJ Health Screening Center, variables including BMI, WC, FG, TG, HDL-C, TT, FT, SHBG, and E2 were measured using automated immunoassay and enzymatic techniques. Specifically, total testosterone (TT) was analyzed via a chemiluminescent microparticle immunoassay on ARCHITECT i2000. Other biochemical markers, including HDL-C (homogeneous direct method), TG (GPO-POD-ESPT method), and FG (HK.G-6-PD.NADP method), were measured using a TOSHIBA C8000 automated chemistry analyzer.

### 2.3 Criteria for definition of hypogonadism and other parameters

Hypogonadism was defined according to the 2018 American Urological Association guideline as TT <300 ng/dL (21). The TyG index was calculated as follows: *TyG index* = ln [*fasting TG* (*mg/dL*) × *fasting glucose (mg/dL)*/2] (5).

Metabolic syndrome (MetS) was defined based on the 2005 American Heart Association/National Heart, Lung, and Blood Institute (AHA/NHLBI) criteria. A diagnosis of MetS in men required the presence of at least three of the following components (22):

1. Waist circumference (WC) ≥90 cm;
2. Triglycerides (TG) ≥150 mg/dL;
3. High-density lipoprotein cholesterol (HDL-C) <40 mg/dL;
4. Blood pressure (BP) ≥130 mmHg systolic or ≥85 mmHg diastolic;
5. Fasting glucose (FG) ≥ 100 mg/dL (22).

Participants were further categorized into four distinct metabolic phenotypes based on their TG and FG levels, in accordance with the 2005 American Heart Association/National Heart, Lung, and Blood Institute guideline for MetS (22):

1. Normal fasting glucose and normal triglycerides (NFGNT): FG < 100 mg/dL and TG < 150 mg/dL;
2. Hypertriglyceridemia (HTG): TG ≥ 150 mg/dL with normal FG;
3. Elevated fasting glucose (EFG): FG ≥ 100 mg/dL with normal TG;
4. Hypertriglyceridemic fasting glycemia (HTFG): Both FG ≥ 100 mg/dL and TG ≥ 150 mg/dL.

### 2.4 Statistical analysis

Data cleaning and preliminary screening were directly performed using IBM SPSS Statistics 22.0 (IBM Corp., Armonk, NY, USA). After importing the raw data from the MJ Health Research Foundation database, the dataset was systematically reviewed for logical inconsistencies and outliers. Participants with missing data on key variables or those who failed to meet the predefined inclusion criteria were excluded in the SPSS environment before the formal analysis.

All analyses were conducted using IBM SPSS Statistics 22.0 (IBM Corp., IBM SPSS Statistics for Windows, Armonk, New York). Continuous variables were expressed as mean ± standard deviation, and categorical variables as frequencies and percentages. In addition, for key metabolic parameters susceptible to skewed distributions (e.g., FG, TG, and TyG index), the median and interquartile range (IQR) are also provided to ensure a robust representation of the data distribution. Group differences in categorical variables were assessed using the chi-squared or Fisher’s exact test, and continuous variables using independent-samples *t* tests or one-way analysis of variance, with Scheffé’s test for *post hoc* comparisons.

Pearson’s correlation was used to evaluate associations between continuous variables. Because the dataset included repeated measurements, generalized estimating equations (GEEs) with appropriate link functions were used to account for within-subject correlations: logistic GEE for categorical outcomes and linear GEE for continuous outcomes. Logistic regression results were expressed as ORs with 95% CIs, and linear regression results as regression coefficients (*β*) with 95% CIs. Univariate and multivariate models were applied. Statistical significance was defined as a two-tailed *p* value <0.05.

### 2.5 Ethics statement

All data were obtained with permission from the MJ Health Research Foundation (authorization code: MJHRF2019016A). The study was approved by the Tri-Service General Hospital Institutional Review Board (TSGHIRB Approval No. A202005160) and conducted in accordance with the Declaration of Helsinki. The conclusions presented herein do not reflect the views of the MJ Health Research Foundation. Written informed consent was obtained from all participants for the use of their data. To ensure anonymity, all personal identifiers were removed. The reporting of this study conforms to the STROBE (Strengthening the Reporting of Observational Studies in Epidemiology) guidelines for retrospective cohort studies, and the STROBE checklist is provided as **Supplementary Material**.

## 3 Results

### 3.1 Abnormal metabolic parameters and high TyG index values are associated with hypogonadism in middle-aged men

From the MJ Health Examination database (2011–2022), 4,269 individuals provided 7,268 examination records, indicating that several participants had multiple visits. Baseline demographic and clinical characteristics are summarized in a **Table 1-1** and **Table 1-2**. Among the 4,269 participants (7,268 records), 11.2% had hypogonadism (testosterone <300 ng/dL). Compared with men without hypogonadism (testosterone ≥300 ng/dL), those with hypogonadism showed a higher prevalence of overweight and obesity, abnormal blood pressure, dyslipidemia, hyperglycemia, and hypertriglyceridemia (all *p* < 0.05).

**Table 1-1:** Baseline biochemical and physiological parameters of the participants.

Characteristics	Testosterone		*p*	Free testosterone	*p*	Total T/E2 ratio	*p*
	≥300 ng/dL (*n* = 3,792)	<300 ng/dL (n = 477)					
FG, mg/dl				−0.075	<0.001^d,***^	−0.077	<0.001^d,***^
mean ± SD	108.37 ± 22.15	117.03 ± 31.84	<0.001^a,***^				
medians (IQR)	104.00 (98.00-111.00)	107.00 (101.11-119.00)	<0.001^b,***^				
TG, mg/dl				−0.053	<0.001^d,***^	−0.075	<0.001^d,***^
mean ± SD	135.10 ± 101.26	176.86 ± 113.74	<0.001^a,***^				
medians (IQR)	111.00 (80.00-162.00)	152.00 (108.00-209.00)	<0.001^b,***^				
TyG index				−0.084	<0.001^d,***^	−0.122	<0.001^c,***^
mean ± SD	8.72 ± 0.60	9.07 ± 0.60	<0.001^a,***^				
medians (IQR)	8.68 (8.33-9.07)	9.05 (8.68-9.44)	<0.001^b,***^				
FG & TG			<0.001^c,***^		<0.001^e,***^		<0.001^e,***^
NFGNT	891 (23.5)	46 (9.6)		10.66 ± 5.39		30.04 ± 33.14	
HTG	250 (6.6)	37 (7.8)		9.73 ± 2.81		21.65 ± 9.74	
EFG	1,804 (47.6)	185 (38.8)		9.90 ± 4.22		25.59 ± 25.85	
HTFG	847 (22.3)	209 (43.8)		9.33 ± 4.93		21.55 ± 25.97	

EFG, FG ≥ 100 mg/dL with normal TG; FG, fasting glucose; HDL, high-density lipoprotein; HTFG, elevated FG and TG; HTG, TG ≥ 150 mg/dL with normal FG; NFGNT, normal FG and TG; TG, triglyceride; triglyceride–glucose index (TyG index) = Ln (FG (mg/dL) × TG (mg/dL)/2).

Continuous variables are presented as mean ± SD and median (interquartile range).

^a^ Independent *t*-test.

^b^ Mann-Whitney U test.

^c^ Pearson’s chi-squared test.

^d^ Pearson’s correlation coefficient.

^e^ One-way ANOVA.

^*^ *p <* 0.05.

^**^ *p <* 0.01.

^***^ *p <* 0.001.

**Table 1-2:** Demographic and general clinical characteristics.

Characteristics	Testosterone		*p*	Free testosterone	*p*	Total T/E2 ratio	*p*
	≥300 ng/dL (*n* = 3,792)	<300 ng/dL (n = 477)					
Age, yr	53.10 ± 4.5	52.44 ± 5.4	0.012^a^	−0.050	<0.001^c,***^	0.032	0.037^*^
BMI			<0.001^b,***^		<0.001^d,***^		<0.001^***^
Normal	1,569 (41.7)	87 (18.4)		10.45 ± 2.98		29.37 ± 30.91	
Underweight	52 (1.4)	0 (0)		11.11 ± 3.63		45.58 ± 61.19	
Overweight	1,394 (37.0)	188 (39.7)		9.85 ± 4.44		23.55 ± 20.66	
Obesity	752 (20.0)	199 (42.0)		9.08 ± 6.77		20.13 ± 25.27	
WC			<0.001^b,***b^		<0.001^b,***^		<0.001^***^
Normal	2,757 (73.2)	241 (50.8)		10.23 ± 3.83		27.25 ± 28.62	
Abnormal	1,009 (23.8)	233 (49.2)		9.19 ± 6.11		20.68 ± 22.71	
BP			<0.001^b,***^		0.007^b,**^		<0.001^***^
Normal	2,161 (59.6)	190 (43.3)		10.11 ± 3.01		26.11 ± 28.40	
Abnormal	1,462 (40.4)	249 (56.7)		9.71 ± 6.29		19.59 ± 13.98	
HDL			<0.001^b,***^		<0.001^b,***^		0.001^**^
Normal	3,378 (89.2)	360 (75.6)		10.02 ± 4.23		26.57 ± 29.63	
Abnormal	410 (10.8)	116 (24.4)		9.15 ± 6.79		23.80 ± 23.81	

BMI, body mass index; BP, blood pressure; WC, waist circumference. BMI, normal (≧18.5, <24), underweight (<18.5), overweight (≧24, <27), and obesity (≧27).

^a^ Independent *t* test.

^b^ Pearson’s chi-squared test.

^c^ Pearson’s correlation coefficient.

^d^ One-way ANOVA.

^*^ *p <* 0.05.

^**^ *p <* 0.01.

^***^ *p <* 0.001.

The TyG index was positively associated with hypogonadism (*p* < 0.001) and inversely correlated with FT (*r* = −0.087, *p* < 0.001) and the total testosterone-to-estradiol ratio (T/E_2_; *r* = −0.122, *p* < 0.001). The prevalence of hypogonadism significantly increased across FG and TG phenotypes: 9.6% in NFGNT, 7.8% in HTG, 38.8% in EFG, and 43.8% in HTFG (*p* < 0.001). FT levels and the total T/E_2_ ratio also differed significantly across these phenotypes (all *p* < 0.001). FT (10.66 ± 5.39 ng/dL) and the T/E_2_ ratio (30.04 ± 33.14) were highest in the NFGNT group, whereas the HTFG group generally showed the lowest values across these indices.

### 3.2 The TyG index is an independent predictor of lower TT and FT levels

After adjustment for measurement time, age, BMI, WC, blood pressure, and HDL, the TyG index remained significantly associated with an increased risk of low testosterone levels (adjusted OR = 1.90; 95% CI, 1.58–2.28; *p* < 0.001). Across the FG and TG phenotypes, the HTFG group had the highest risk of low testosterone levels (adjusted OR = 2.55; 95% CI, 1.86–3.51; *p* < 0.001), followed by the HTG (adjusted OR = 2.16; 95% CI, 1.48–3.17; *p* < 0.001) and EFG (adjusted OR = 1.61; 95% CI, 1.19–2.16; *p* = 0.002) groups, compared with the NFGNT reference group (**Table 2**).

**Table 2:** Multivariable-adjusted GEE model of the TyG index and the FG and TG categories with hypogonadism.

Variable	Adjusted OR	95% CI	
		Lower	Upper
Adjusted model for TyG index	1.90^***^	1.58	2.28
Adjusted model for FG and TG			
NFGNT	Ref.		
HTG	2.16^***^	1.48	3.17
EFG	1.61^**^	1.19	2.16
HTFG	2.55^***^	1.86	3.51

CI, confidence interval; EFG, FG ≥100 mg/dL with normal TG; FG, fasting glucose; GEE, generalized estimating equations; HTFG, elevated FG and TG; HTG, TG ≥150 mg/dL with normal FG; NFGNT, normal FG and TG; OR, odds ratio; TG, triglyceride; TyG, triglyceride–glucose.

The adjusted model for the TyG index (red point) included adjusted measurement time, age, BMI, WC, BP, HDL-C, and the TyG index difference. Adjusted model for FG & TG (blue point) was adjusted for measurement time, age, BMI, WC, BP, and HDL-C.

^*^ *p* < 0.05.

^**^ *p <* 0.01.

^***^ *p <* 0.001.

The TyG index was significantly and inversely associated with FT (*β* = −0.294; 95% CI, −0.501 to −0.087; *p* = 0.005) (**Table 3**). Consistent patterns were observed across the FG and TG phenotypes. The HTFG group exhibited the greatest reduction in FT (*β* = −0.530; 95% CI, −0.963 to −0.097; *p* = 0.017), followed by the HTG group (*β* = −0.465; 95% CI, −0.929 to −0.001; *p* = 0.049). The EFG group demonstrated a moderate but not significant reduction in FT (*β* = −0.254; 95% CI, −0.693 to 0.185; *p* = 0.256). These associations remained robust after multivariable adjustment.

**Table 3:** Multivariable-adjusted GEE model of free testosterone.

Independent Variables	Adjusted model for TyG index				Adjusted model for FG and TG			
	*ß*	*SE*	95% CI		*ß*	*SE*	95% CI	
			Lower	Upper			Lower	Upper
Measurement time	−0.014	0.054	−0.119	0.092	−0.013	0.053	−0.118	0.091
Age, years	−0.020	0.041	−0.101	0.061	−0.018	0.040	−0.096	0.061
BMI								
Normal	Ref.				Ref.			
Underweight	0.723	0.511	−0.279	1.725	0.762	0.508	−0.232	1.757
Overweight	−0.576^*^	0.276	−1.118	−0.035	−0.572^*^	0.281	−1.123	−0.021
Obesity	−0.880^**^	0.273	−1.415	−0.344	−0.874^**^	0.284	−1.431	−0.317
WC								
Normal	Ref.				Ref.			
Abnormal	−0.529^***^	0.144	−0.812	−0.246	−0.538^***^	0.146	−0.823	−0.253
BP								
Normal	Ref.				Ref.			
Abnormal	0.015	0.249	−0.474	0.504	0.017	0.247	−0.468	0.502
HDL								
Normal	Ref.				Ref.			
Abnormal	−0.725^**^	0.237	−1.189	−0.262	−0.752^**^	0.237	−1.216	−0.288
TyG index	−0.294^**^	0.106	−0.501	−0.087				
FG & TG								
NFGNT					Ref.			
HTG					−0.465^*^	0.237	−0.929	−0.001
EFG					−0.254	0.224	−0.693	0.185
HTFG					−0.530^*^	0.221	−0.963	−0.097

CI, confidence interval; EFG, FG ≥100 mg/dL with normal TG; FG, fasting glucose; GEE, generalized estimating equations; HDL, high-density lipoprotein; HTFG, elevated FG and TG; HTG, TG ≥150 mg/dL with normal FG; NFGNT, normal FG and TG; OR, odds ratio; TG, triglyceride; TyG, triglyceride–glucose.

Adjusted variables include measurement time, age, BMI, WC, BP, and HDL-C.

^*^ *p* < 0.05.

^**^ *p <* 0.01.

^***^ *p <* 0.001.

### 3.3 Longitudinal changes in TyG are associated with discordant changes in testosterone levels

A subgroup analysis (*n* = 3,792) restricted to men with baseline testosterone ≥300 ng/dL assessed whether longitudinal changes in the TyG index predicted the development of hypogonadism. Participants with an increase in TyG index (>0) had a significantly higher likelihood of developing hypogonadism compared with those whose TyG index decreased or remained stable (≤0; OR = 1.957; 95% CI, 1.527–2.508; *p* < 0.001) (**Table 4**; Model I).

**Table 4:** GEE subgroup^a^ analysis of TyG index changes and risk of hypogonadism.

Testosterone ≧300 ng/dL								
Models	Changes in TyG index	Observations	OR	*p*	95% CI	
					Lower	Upper
Model I	Changes in TyG index					
	≦0	5,428	Ref.			
	>0	1,355	1.957	<0.001^***^	1.527	2.508
Model II	Changes in TyG index					
	<0.5	6,552	Ref.			
	≧0.5	231	2.077	0.004^**^	1.263	3.417
Model III	Changes in TyG index					
	≦0	5,428	Ref.			
	>0, <0.5	1,124	1.871	<0.001^***^	1.439	2.433
	≧0.5	231	2.380	0.001^**^	1.436	3.943

CI, confidence interval; GEE, generalized estimating equations; OR, odds ratio; TyG, triglyceride–glucose.

^a^ Subgroup analysis was restricted to participants with baseline testosterone levels ≥300 ng/dL.

^*^ *p* < 0.05.

^**^ *p <* 0.01.

^***^ *p <* 0.001.

In Model II, using a higher cutoff, participants with ΔTyG ≥0.5 had an approximately twofold higher risk of incident hypogonadism (OR = 2.077; 95% CI, 1.263–3.417; *p* = 0.004). Model III further demonstrated a dose–response pattern. Compared with men whose TyG index decreased or remained stable (values ≤0), those with a modest increase (0 < TyG < 0.5) had an elevated risk (OR = 1.871; 95% CI, 1.439–2.433; *p* < 0.001), and the risk was highest among those with increases ≥0.5 (OR = 2.380; 95% CI, 1.436–3.943; *p* = 0.001) (**Table 4**).

The subgroup analysis in participants with baseline testosterone <300 ng/dL (*n* = 477) did not identify significant associations between ΔTyG and subsequent testosterone levels (**Table 5**). In Model I, no significant difference was observed between individuals with stable or decreased TyG index (≤0) and those with increased values (>0; *β* = 12.99; 95% CI, −5.05 to 31.03). In Model II, decreases in the TyG index ≤−0.5 (*β* = −2.04; 95% CI, −34.33 to 30.24) or between −0.5 and 0 (*β* = 13.57; 95% CI, −4.50 to 31.63), relative to the increased group (>0), showed directional but not significant trends (*p* > 0.05). Model III revealed similar nonsignificant patterns, with participants demonstrating declines between −0.1 and −0.5 (*β* = −21.70; 95% CI, −45.06 to 1.65) or declines ≤−0.5 (*β* = −15.95; 95% CI, −43.90 to 12.00) tending to show lower testosterone levels compared with those with increased TyG values (**Table 5**).

**Table 5:** GEE subgroup^a^ analysis of TyG index changes and risk of hypogonadism.

Testosterone <300 ng/dL								
Models	Changes in TyG index	Observations	*ß*	*SE*	95% CI	
					Lower	Upper
Model I	Changes in TyG index					
	≦0	436	12.99	9.20	−5.05	31.03
	>0	49	Ref.			
Model II	Changes in TyG index					
	≦−0.5	16	−2.04	16.47	−34.33	30.24
	>−0.5, ≦0	420	13.57	9.22	−4.50	31.63
	>0	49	Ref.			
Model III	Changes in TyG index					
	≦−0.5	16	−15.95	14.26	−43.90	12.00
	>−0.5, <0	38	−21.70	11.92	−45.06	1.65
	≧0	431	Ref.			

CI, confidence interval; GEE, generalized estimating equations; OR, odds ratio; TyG, triglyceride–glucose.

^a^ Subgroup analysis was restricted to participants with baseline testosterone levels <300 ng/dL.

## 4 Discussion

This study of Taiwanese men aged 45–65 years from the MJ Health Examination database demonstrated that a higher triglyceride-glucose (TyG) index was inversely associated with serum total and free testosterone (FT) levels, independent of age, body mass index (BMI), waist circumference (WC), blood pressure (BP), and high-density lipoprotein (HDL). Longitudinal changes in the TyG index were further associated with a higher risk of developing hypogonadism among men with baseline testosterone ≥300 ng/dL, showing a clear dose–response relationship. Stratified analysis revealed that, compared with the normal fasting glucose and normal triglycerides (NFGNT) group, the hypertriglyceridemic fasting glycemia (HTFG) group had the highest risk of low testosterone level (OR = 2.55), followed by the hypertriglyceridemia (HTG) (OR = 2.16) and elevated fasting glucose (EFG) (OR = 1.61) groups. These findings suggest that triglyceride (TG) levels exert a stronger influence than glucose levels on the development of hypogonadism in middle-aged men, underscoring the importance of maintaining optimal lipid metabolism to preserve testosterone levels.

Previous cohort studies have indicated that persistent or increasing TyG-related variables reflect worsening insulin resistance (IR) and metabolic stress, both of which substantially increase the risk of cardiovascular disease (CVD) and type 2 diabetes mellitus (T2DM) (23, 24). However, none of these studies discussed hypogonadism. To the best of our knowledge, this is the first study to examine longitudinal changes in the TyG index itself, rather than TyG-related composite measures, and their association with incident hypogonadism in previously nonhypogonadal individuals.

The TyG index is widely recognized as a surrogate marker of IR in apparently healthy individuals (5) and as a simple measure of insulin sensitivity when compared with the euglycemic–hyperinsulinemic clamp test (6). It has also been validated in patients with metabolic syndrome (MetS), arterial stiffness, early-stage chronic kidney disease, and T2DM (25–27). Several studies have compared the performance of the TyG and homeostatic model assessment of insulin resistance (HOMA-IR) indices in predicting male hypogonadism (28). Although some evidence suggests that the TyG index is not superior to HOMA-IR for identifying testosterone deficiency among adult men (29), it remains a practical and reliable surrogate of IR. Its strong association with the risk of developing hypogonadism highlights its potential clinical utility, particularly in settings where fasting insulin measurements for HOMA-IR are unavailable (29).

In the present study, the mean TyG index was 8.72 ± 0.60 in men with baseline testosterone ≥300 ng/dL and 9.07 ± 0.60 in those with testosterone <300 ng/dL. These findings are consistent with those of Yilmaz et al., who reported that men with TyG >8.88 had a substantially higher risk of erectile dysfunction (OR = 3.865; 95% CI, 1.686–8.859) (30), reinforcing the TyG index as a clinically relevant marker of metabolic and sexual function impairment.

We further stratified the TyG index into its fasting glucose (FG) and TG components, generating four phenotypes: NFGNT, HTG, EFG, and HTFG. Compared with NFGNT, the HTFG group had the highest risk of low testosterone, followed by the HTG and EFG groups. This gradient suggests that TG plays a more influential role than FG in testosterone suppression. This interpretation is consistent with reports that high TG and low high-density lipoprotein cholesterol (HDL-C) levels are more strongly associated with visceral adiposity than FG (31). Visceral fat accumulation enhances aromatase activity and stimulates inflammatory cytokine production, which suppress gonadotropin release and testosterone synthesis (32). Contrarily, FG primarily reflects short-term glycemic conditions and may not adequately capture the chronic metabolic burden contributing to androgen decline. Thus, TG may serve as a more sensitive biomarker linking visceral obesity to hypogonadism.

The biological mechanisms linking elevated TyG to testosterone deficiency are likely multifactorial. An abnormal TyG index can also indicate abnormal serum lipid profiles (dyslipidemia), which promotes the release of proinflammatory cytokines (e.g., interleukin-6 [IL-6] and tumor necrosis factor-alpha [TNF-α]) from endothelial cells and monocytes (33). In a previous animal study, male rabbits fed a high-fat diet developed MetS accompanied by significant hypogonadism and elevated interleukin-1 beta (IL-1β), IL-6, and TNF-α levels (34). TNF-α has also been shown to inhibit steroidogenesis in Leydig cells by downregulating steroidogenic enzyme transcription (35). These mechanisms collectively support the observed association between elevated TyG levels and testosterone decline.

In conclusion, our findings suggest that longitudinal increases in the TyG index may serve as a convenient and cost-effective clinical indicator for predicting testosterone deficiency, particularly in resource-limited settings where direct hormone assays are unavailable. Because fasting TG and glucose levels are routinely measured in clinical practice, the TyG index could be readily integrated into screening algorithms. Clinicians should consider evaluating testosterone levels in men with high TyG values (e.g., ≥8.83) who present with symptoms such as fatigue, decreased libido, or depression, and assess whether testosterone replacement therapy may be appropriate (29).

### 4.1 Strengths and limitations

This study has several strengths. First, it included a relatively large sample of Taiwanese men (n = 4,269), ensuring robust statistical power and enhancing representativeness within an Asian population. Second, the repeated measure’s longitudinal design enabled the assessment of within-individual changes in testosterone levels over time. This design minimizes inter-individual variability and strengthens causal inference by revealing dynamic associations between metabolic indices and androgen decline. In contrast, previous studies, such as those by Liu et al. (10) and Zhang et al. (19), relied on cross-sectional data collected at a single time point. Existing evidence relies on cross-sectional datasets, such as NHANES, which lack the temporal depth to establish causality. Contrarily, the MJ database in Taiwan provides high-quality longitudinal data with repeated measurements. By using generalized estimating equations (GEEs), this study can account for within-subject correlations, thereby providing a more dynamic and robust analysis of how metabolic fluctuations drive testosterone decline in an East Asian context.

However, several limitations must be acknowledged. First, participants at the MJ Health Screening Center are self-paying individuals who voluntarily undergo health examinations, likely representing a population with higher socioeconomic status and greater health awareness. This may limit the generalizability of our findings to the broader population. Second, the database lacks detailed clinical information, including physician-confirmed diagnoses and specific symptom profiles, which may lead to incomplete health status assessments and potential residual confounding. Third, participants could choose the timing and frequency of their examinations, potentially introducing selection bias. Future studies incorporating more comprehensive clinical data and interventional designs are needed to further clarify the causal mechanisms underlying these associations.

## 5 Conclusions

Our findings indicate that the TyG index is a dynamic and practical biomarker for identifying men at increased risk of androgen deficiency. Increases in TyG levels were independently associated with a higher risk of hypogonadism and lower FT levels. Individuals with persistently elevated TyG levels, particularly those in the HTFG phenotype, exhibited the greatest susceptibility to hypogonadism.

## Data Availability

The dataset is subject to legal and ethical restrictions under Taiwan’s Personal Information Protection Act and is managed by the MJ Health Research Foundation. Public access is not permitted, and data access requires formal application and approval. Requests to access the datasets should be directed to MJ Health Research Foundation Website: https://www.mjhrf.org Data access requests should be submitted through the official application process described on the website.
